# How oral health care organizations formulate actionable statements to inform practice and policy: A protocol for a systematic survey

**DOI:** 10.12688/f1000research.141423.1

**Published:** 2023-10-03

**Authors:** Francisca Verdugo-Paiva, Xavier Bonfill Cosp, Pablo Alonso-Coello, Camila Ávila-Oliver, Michael Glick, Alonso Carrasco-Labra

**Affiliations:** 1 Epistemonikos Foundation, Santiago, Santiago Metropolitan Region, Chile; 2 Programa de TTM y Dolor Orofacial, Facultad de Odontología, Universidad Andres Bello, Santiago, Santiago Metropolitan Region, Chile; 3 Department of Paediatrics, Obstetrics, Gynaecology and Preventive Medicine and Public Health, Universitat Autonoma de Barcelona, Barcelona, Catalonia, Spain; 4 Clinical Epidemiology Service, Hospital Sant Pau, Barcelona, Spain; 5 Iberoamerican Cochrane Centre, Sant Pau Biomedical Research Institute, Barcelona, Spain; 6 Escuela de Odontología, Facultad de Odontología y Ciencias de la Rehabilitación, Universidad San Sebastián, Santiago, Santiago Metropolitan Region, Chile; 7 Center for Integrative Global Oral Health, School of Dental Medicine, University of Pennsylvania, Philadelphia, Pennsylvania, USA

**Keywords:** Guidelines; guidance; Recommendations; GRADE; Practice statements; Policy; Evidence-to-decision framework; Evidence-Based Dentistry, Oral Health policy

## Abstract

**Background:** Oral diseases are a major global public health problem that impacts the quality of life of those affected. While widespread consensus exists on the importance of high-quality, evidence-informed guidelines to inform practice and public health decisions in medicine, appropriate methodologies and standards are not commonly adhered to among producers of oral health guidelines. This systematic survey aims to identify organizations developing evidence-informed guidelines and policy documents in oral health globally, and describe the methods and processes used.

**Methods:** We will conduct manual searches on the websites of guideline developers, Ministries of Health, and scientific societies. Additionally, we will systematically search electronic databases to identify published guidelines and collect the name of the responsible entity. We will include organizations that regularly develop guidelines on any oral health topic and that explicitly declare the inclusion of research evidence in its development process. Subsequently, we will use a standardized form to extract data about the characteristics of the organization, the characteristics of their guideline or policy documents, and their formal recommendation development processes. These data will be extracted from various sources, such as the organization’s official website, the methods section of each guideline, or methodological handbooks. We will use descriptive statistics to analyze the extracted data.

**Discussion:** This systematic survey will synthesize key characteristics and methodologies used by organizations developing evidence-informed guidelines. This study will provide the basis for future development of a sustainable and connected collaborative network for evidence-informed guidelines and policy documents in oral health globally. The results will be disseminated through peer-reviewed publications, conference presentations, and targeted dissemination of findings with the identified organizations. Our systematic survey represents a necessary first step toward improving the field of oral health policies and guidelines.

## Background

Guidelines are systematically developed evidence-informed statements, including recommendations for clinical practice or public health policy. ^1^^,^^2^ Guidelines are fundamental to translate and transfer scientific knowledge to patients, caregivers, clinicians, policy-makers, and other decision-makers. ^3^ Ultimately, the optimal development, dissemination, and implementation of high-quality guidelines may improve the performance of health systems and enhance health outcomes. ^4^

Recommendations or actionable statements contained in guidelines should be developed using systematic and transparent methods to identify and synthesize available evidence, as well as engage stakeholders and agreed upon recommendations. ^5^ In the medical field, there is a wide consensus about well-conducted systematic review being the most reliable approach to synthesizing trustworthy evidence, and a considerable number of frameworks for addressing evidence to decision process are available, ^6^ such as the GRADE-EtD framework ^7^ (Grading of Recommendations Assessment, Development, and Evaluation - Evidence to Decision).

Multiple organizations worldwide, including scientific societies, professional associations, and Ministries of Health, produce guidelines to support dental practice and inform public oral health decisions. However, these organizations rarely follow appropriate methodologies, leading to guidelines with suboptimal quality. ^8^^–^^12^ Despite the availability of standards and definitions for the different types of actionable statements contained in guidelines, policy guidance, and similar standards-setting documents in medicine, ^3^ there is a vast set of terminologies used to categorize these documents among organizations developing evidence-informed guidelines in oral health globally. Such misclassification could hinder interprofessional communication and medical and dental care integration.

Developing high-quality healthcare guidelines is a complex and time-consuming process. ^2^ However, there is growing demand from stakeholders globally in oral health for these products. In 2022, the World Health Organization officially approved and adopted the Global oral health strategy, which states that evidence-informed policies of cost-effective interventions must be developed and implemented to influence global and national oral health outcomes. The document also highlighted the importance of translating research findings into practice, including the development of regionally specific, evidence-informed guidance. ^13^

Oral diseases are a global public health problem and decrease the quality of life and well-being of those affected. As the most prevalent diseases, such as dental caries, periodontal diseases, and oral cancer, are mostly preventable; high-quality guidelines to address oral diseases with evidence-informed, cost-effective, and safe interventions are much needed. ^14^ To make the needed improvement to ensure that high-quality guidelines are available for all relevant oral health conditions, this systematic survey is a first step towards knowing the organizations that generate these documents worldwide and comprehensively understanding how such guidelines are been developed.

## Objectives

This systematic survey aims to identify organizations developing guidelines in oral health globally, and describe methods and processes used to formulate actionable statements.

## Methods

### Eligibility criteria

We will include organizations that regularly develop guidelines in oral health, such as scientific societies, Ministries of Health, professional associations, non-governmental organizations, or any working group. For the purpose of this study, we will consider a ‘guideline’ any document or information product containing actionable statements that recommends or suggests a particular course of action for clinical practice or public health policy. ^1^^,^^2^

Within the term ‘guideline’, we will consider clinical practice guidelines, guidances, and similar policy documents that enhance the decision-making process, by translating research findings into actionable statements for healthcare practice, public health or policy decisions at the local (national or sub-national level), regional, or global level, and for a diverse group of stakeholders, including but not restricted to patients, healthcare professionals, researchers, institutions, or organizations. ^1^^,^^2^^,^^4^^,^^5^^,^^15^

To be included, organizations worldwide must fulfill the following criteria:

• Produce at least three guidelines on any oral health topic since 2012, according to the oral health definition provided by the FDI World Dental Federation and the WHO. ^16^^,^^17^
• Produce guidelines that explicitly declare the inclusion of research evidence in its development processes, regardless of whether the organization performs a *de novo* systematic review, uses pre-existing systematic review, or conducts non-systematic literature reviews to support its decisions.

Organizations that solely produce educational documents or health system policy documents (documents containing actionable statements related to service delivery, health workforce, health information systems, access to essential medicines, vaccines, technology, financing, and leadership or governance) will be excluded.

### Search and selection of the eligible organizations

To identify organizations responsible for guideline development in the field of oral health globally, we will perform both a systematic and manual search. First, we will systematically search for oral health guidelines in electronic databases (PubMed, Epistemonikos database) and guideline repositories, including the Clinical Practice Guidelines (CPG) Infobase, the International Guidelines Library from the Guideline International Networks (GIN), the Guideline Central, the Alliance for the Implementation of Clinical Practice Guidelines (AiCPG) and the Medical Information Distribution Service (Minds) database. We will not limit the search by language or publication status, but the date will be restricted (2012-present). Two reviewers will independently evaluate whether the documents identified are eligible, according to our definition of a guideline. Any disagreements will be resolved with discussions between the independent reviewers. If consensus cannot be reached, a third reviewer (FV-P), will resolve disagreements. The organizations’ names could be collected from various sources, such as the corresponding authors’ information, the supporter/funder organization, or the methods section of the identified document.

In parallel, we will perform a manual search on the websites of guideline developers, scientific societies, professional associations, and ministries of health globally. In addition, we will consult with experts in the field to identify missing organizations that meet the inclusion criteria.

**Figure 1.**
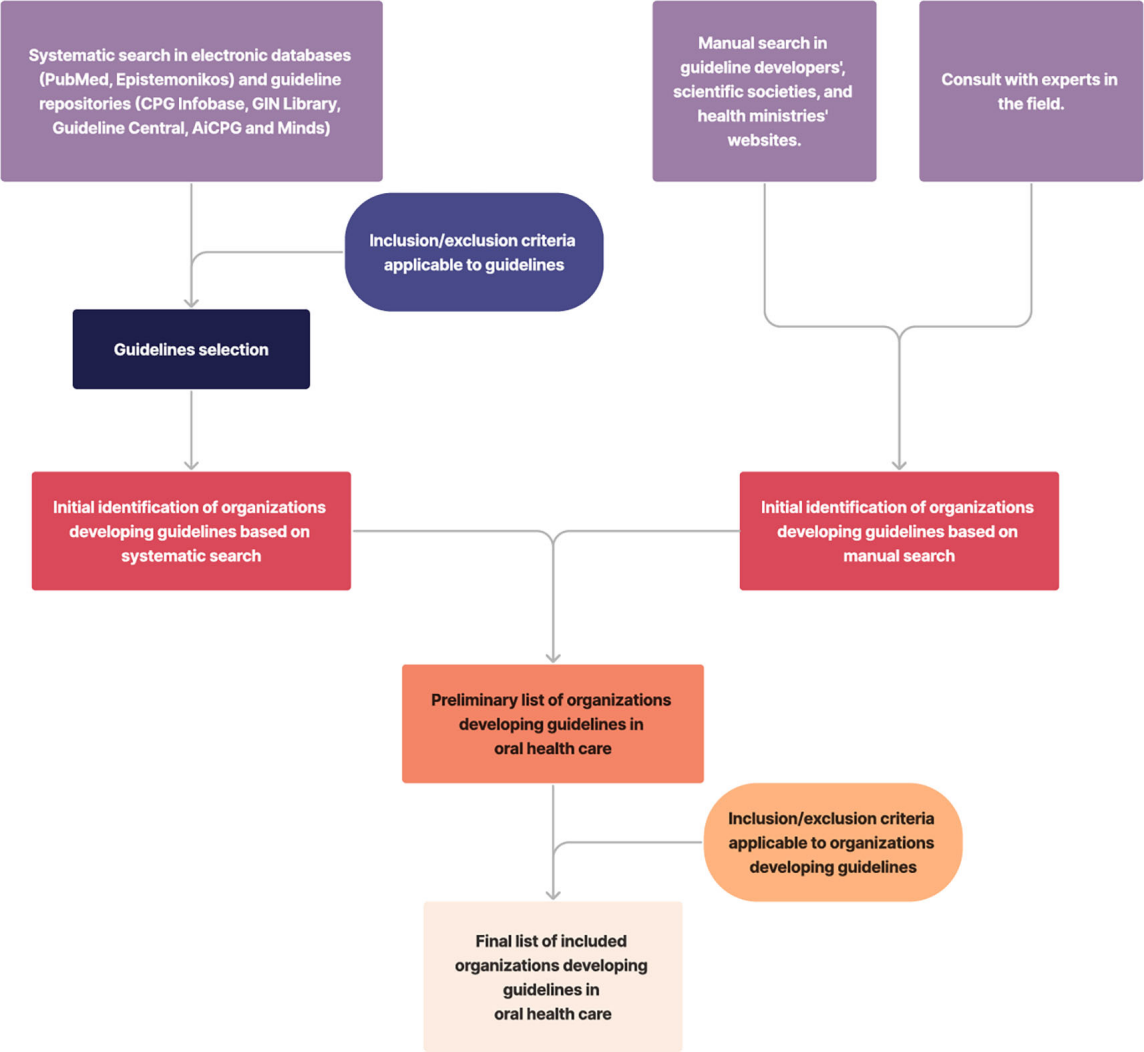
Flowchart of search, identification, and selection of organizations producing guidelines.

Two reviewers will independently evaluate whether the organizations detected by systematic or manual search are eligible for inclusion in this study, according to the predefined criteria outlined above. The Figure 1 shows the flowchart of the organization’s search and selection. The electronic databases search strategy, the sources used, and the consulted websites are listed in the extended data (Extended data 1).

### Data extraction

**Figure 2.**
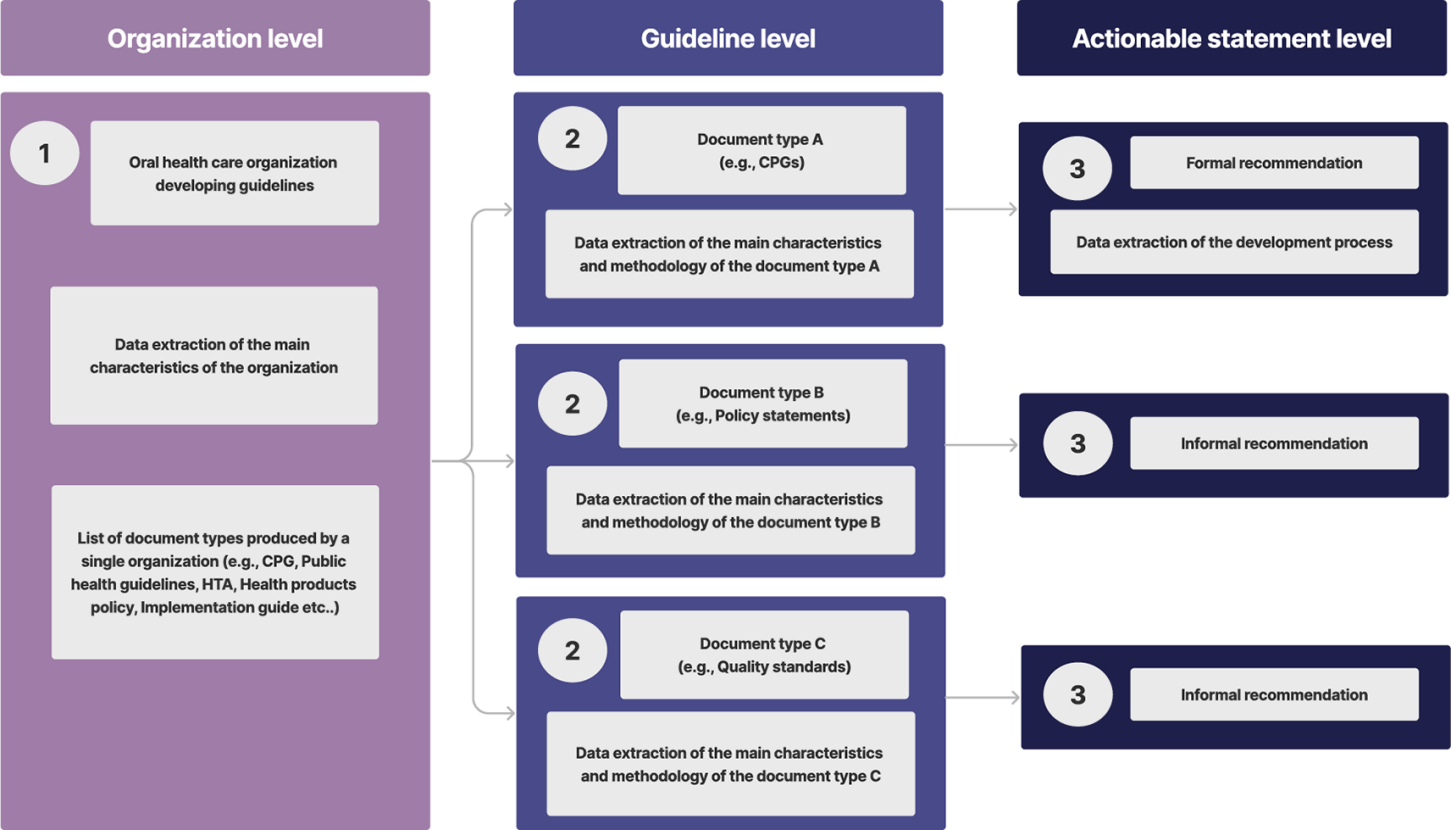
Phases of data extraction.

After an initial calibration exercise, two trained reviewers will extract data independently, using a previously piloted standardized form. Calibration will consist of performing the complete data extraction of two different organizations. Disagreements will be solved through discussion and consensus or with the help of a third reviewer. The data extraction will be divided into three phases: a) Organization level, b) Guidelines or policy documents level, and c) Actionable statement level ( Figure 2).

1) **Organization level**
  The form will cover information on the main characteristics of the included organizations, including the organization type (e.g, non-governmental organization, governmental organization, or academic and research institution), country, language, oral health clinical specialty, number of documents produced over the last ten years, and the types of documents produced. The oral health clinical specialty will be classified according to the list and definition of the recognized dental specialties approved by the National Commission for the Recognition of Dental Specialties and Certification Boards of the American Dental Association (ADA) ^18^ and the European recognized dental specialties. ^19^ Regarding the types of documents, we will extract the document name and their description, according to the information provided by the organization (e.g, an organization produces “Clinical Guidelines”, defined as recommendations for clinical practice based on a systematic review of the evidence, along with an assessment of the benefits and possible harms of alternative dental care options, and the same organization also produces “Oral Health Policies”, defined as evidence-informed statements relating to the organization positions on various public health issues). We will categorize these documents into the following categories: 1. Clinical practice guidelines, 2. Public health guidelines, 3. Health products policy and standards (including Health Technology Assessment and Policy statements), and 4. Other, according to the following definitions:
**Guidelines:** Corresponds to any product that includes systematically developed statements recommending or suggesting a particular course of action for clinical practice or public health decisions. Recommendations are actionable statements designed to help end-users make informed health decisions related to clinical interventions, diagnostic tests, or public health measures to achieve the best individual or collective health outcomes. ^1^^,^^2^^,^^5^
**Policy statements:** evidence-informed statements addressing issues of concern and importance to the public health community. “ *Policy statements should describe and endorse a defined course of action, ranging from legislation and regulations desired to needed new policies of non-governmental organizations and private enterprises”.* ^20^
**Health technology assessment: “** *Health technology assessment (HTA) refers to the to the systematic evaluation of health technology’s properties, effects, and/or impacts. It is a multidisciplinary process to evaluate the social, economic, organizational, and ethical issues of a health intervention or health technology. The main purpose of conducting an assessment is to inform a policy decision-making. Considering the definition of health technology, as the application of organized knowledge and skills in the form of medicines, medical devices, vaccines, procedures, and systems developed to solve a health problem and improve quality of life”.* ^21^
2) **Guidelines or policy documents level**
  For each document type produced by a single organization, we will extract the information about the main characteristics of the guideline documents, as well as characteristics of the methodology, including the intended users, stakeholders’ involvement, information about the working group or panel composition, conflicts of interest (COI) management policy, and sources of funding. For example, if an organization produces more than one document type with a distinct methodology (for example, a Ministry of Health produces clinical practice guidelines and policy statements), we will extract the data for each document type independently. If this information has changed over the years, we will extract the data from the latest published document.
  Finally, we will use the taxonomy developed by Lofti et al. to classify the statements types used within guidelines, to determine which guideline or policy document contains formal recommendations. A formal recommendation is an actionable statement about the selection between two or more interventions in a specific population and, if applicable, in a particular setting. These statements are the results of a structured process, and they are explicitly linked to the evidence resulting from a systematic literature search and appraisal process. ^3^
3) **Actionable statement level**
  We will extract the development process information of the formal recommendations contained in the guidelines. The extraction form will cover information on the type of methodological handbook used (e.g., International organization handbook, In-house handbook), the methods for searching and identifying the research evidence (e.g., systematic review, non-systematic literature review), and information about the evidence-to-decision (EtD) process, including the use of frameworks (e.g., GRADE-EtD, the Scottish Intercollegiate Guidelines Network framework, the United States Preventive Services Task Force framework). A framework is defined as any structure of concepts underlying a structured process, in this case, the process of formulating recommendations (EtD process). ^6^
  The information from each organization, the document type, and the formal recommendation development processes will be collected from various sources, such as the organization’s official website, the methods section of each guideline, a reference manual, or a methodological handbook. If available, we will include the latest version of the methodological handbook. If the methods for developing formal recommendations have changed over the years, we will extract the data from the latest published document.

Tables 1, 2, and 3 show the characteristics that will be extracted for each organization and document type.

**Table 1.** Organization level - Main characteristics.

Organization type	Continent	Language	Dental specialty	Number of documents produced over the last ten years	Document type
**1. Non-governmental organization**	Asia	English	Dental Anesthesiology	3-5	**1. Clinical practice guidelines**
1.1 Scientific society	Africa	Mandarin Chinese	Dental Public Health	6-10	**2. Public health guidelines**
1.2 Professional association	Europe	Hindi	Endodontics	11-15	**3. Health products policy and standards**
**2. Governmental Organization**	North America	Spanish	Oral and Maxillofacial Pathology	≥16	3.1 Health technology assessment
2.1 Ministry of Health	Middle America	French	Oral and Maxillofacial Radiology		3.2 Policy statements
2.2 Governmental healthcare agency	South America	Arabic	Oral and Maxillofacial Surgery		**4. Other**
**3. Academic and research institutions**	Australia/Oceania	Bengali	Oral Medicine		
3.1 University	Antarctica	Russian	Orofacial Pain		
3.2 Research center	NA	Portuguese	Orthodontics and Dentofacial Orthopedics		
		German	Pediatric Dentistry		
		Other	Periodontics		
			Prosthodontics		
			Restorative		
			Special Care		
			Stomatognathic physiology		
			General Dentistry		

**Table 2.** Guideline level - Main characteristics and methodology.

Guideline or policy level	Intended users *	Stakeholder involvement	COI policy	COI reported	Funding source
Local (sub-national)	Policy-makers	Clinicians	**COI management policies**	**Non-financial interest only**	**1. Funding source reported**
National	Healthcare managers	Methodologists	**No COI management policies**	**Financial interests only**	1.1 Non-profit association
Regional	Organizational leaders	Policy-makers	**Not reported**	**Financial and non-financial interests**	1.2 Government
Global	Healthcare professionals	Patient representatives			1.3 Industry
	Researchers	Others			1.4 Medical Association
	Citizens				**2. No reported**

COI: conflicts of interest.

* More than one response is possible.

**Table 3.** Actionable statement level - Methods and processes used for formal recommendation development.

Methodological handbook	Methodology to identify evidence	Methodology to assess the certainty of evidence *	Approach for deciding the direction and/or grading the strength *	Frameworks used for EtD process *
**1. Handbook used**	**1. Formal SR**	**1. Formal assessment**	**1. Formal approach**	**1. Formal EtD framework**
1.1 International organization handbook (e.g., WHO)	1.1 *De novo* SR	1.1 GRADE	1.1 GRADE	1.1 GRADE-EtD
1.2 Guidelines development methodology (e.g., GRADE- ADOLOPMENT)	1.2 Previous SR	1.2 OCEBM Levels of Evidence	1.2 NHMRC	1.2 NICE
1.3 In-house handbook	1.3 Previous guidelines or policy	1.3 NHMRC	1.3 SIGN	1.3 SIGN
**2. No handbook used**	**2. Non-systematic literature review**	1.4 SIGN	1.4 CEBM	1.4 USPSTF
**3. Unclear**	**4. Unclear**	1.5 USPSTF	1.5 NICE	1.5 Own approach
		1.6 Own approach	1.6 USPSTF	1.6 Other
		1.7 Other	1.7 Own approach	**2. Unclear**
		**2. Unclear**	1.8 Other	
			**2. Unclear**	

SR: Systematic Review; EtD: Evidence to Decision; GRADE: Grading of Recommendations Assessment, Development, and Evaluation; OCEBM: Oxford Centre for Evidence-Based Medicine; NHMRC: Australian National Health and Medical Research Council; SIGN: Scottish Intercollegiate Guidelines Network; USPSTF: United States Preventive Services Task Force; NICE: National Institute for Health and Care Excellence; GRADE-EtD: Grading of Recommendations Assessment, Development, and Evaluation Evidence to Decision.

* The list of options is based on the results reported by Meneses-Echavez et al. ^6^ We will add new options if new approaches and frameworks are used in the oral health field.

### Data analysis and synthesis

The results and data will be analyzed using descriptive statistics, including mean and median and their corresponding measures of dispersion. Frequencies and proportions will be calculated for all variables. We have identified a series of taxonomies, for example, to classify how the organizations describe their methodology to assess the certainty of the evidence, the determination of the direction and strength of actionable statements, and frameworks to move from the evidence to the decisions. These taxonomies will be reviewed and updated in an iterative process as new categories emerge ( Tables 2 and 3). Data will be presented in text and tables using narrative synthesis.

We will use the synthesized quantitative and qualitative data to produce a series of recommendations to improve the production of oral health guidelines worldwide. These recommendations will be formulated in a panel meeting including experts in the conduct of evidence synthesis and the creation of guidelines and policies. This group will include epidemiologists, statisticians, methodologists with expertise in systematic reviews and meta-analysis, experts trained in the use of the GRADE approach, investigators with expertise in patients’ values and preferences elucidation, policymakers, and a patient partner member.

## Discussion

Scant information is available about how guidelines are being developed in the oral health field worldwide. Guidelines and policy documents available are dispersed in different sources, and there is a lack of coordination and dialogue among the different institutions, leading to wasted resources.

This systematic survey will synthesize the main characteristics and the methodology used by organizations dedicated to guideline and policy development to formulate actionable statements in oral health. This is the first study that seeks to identify the organizations responsible for developing oral health policies and guidelines worldwide, as well as describe the processes and methods followed.

By summarizing this information, we will gain insight for future assessment of potential barriers to and facilitators for using research evidence and applying EtD frameworks in these documents. This study will provide a foundation for the future development of a sustainable and connected collaborative network for evidence-informed guidelines and policies in oral health globally. The results will be disseminated through peer-reviewed publications, conference presentations, and targeted dissemination of findings with the identified organizations and other stakeholders in the area of clinical and policy decision-making. Our systematic survey represents an important first step toward informing the adoption and creation of standardized processes for the development of oral health policies and guidelines that meet current methodological standards in the medical field. This goal further contributes to the dental and medical integration paradigm.

### Ethics and dissemination

No ethical approval is needed for this systematic survey. The authors intend to present the findings at target conferences and publish the research findings in a peer-reviewed journal adopting open science practices.

### Study status

Search strategy completed. Initiating data collection. Analysis expected to be completed by December 2023.

#### Patient and public involvement

No patient and public involvement took place in the development or conduct of this protocol.

## Data Availability

No data are associated with this article. **Fighshare:** Guideline and policy developers in Oral health care. Search strategy and sources, https://doi.org/10.6084/m9.figshare.23995140.v1. ^22^ Data are available under the terms of the Creative Commons Attribution 4.0 International license (CC-BY 4.0).
