# Supplementary material for: How oral health care organizations formulate actionable statements to inform practice and policy: A protocol for a systematic survey
Source: F1000Res. 2023 Oct 3;12:1261. [Version 1] doi: 10.12688/f1000research.141423.1 (PMC10656514; doi:10.12688/f1000research.141423.1)
Supplement: Peer review history

# Reviewer report: Reviewer response for version 1

**DOI:** 10.5256/f1000research.154867.r216660

**Published:** 2023-11-17

**Authors:** Douglas Stirling

**Affiliations:** 1 SDCEP, NHS Education for Scotland, Dundee, Scotland, UK; 2 Dundee Dental Hospital and Research School, University of Dundee, Dundee, Scotland, UK

The authors propose a comprehensive investigation of the methods used to develop actionable statements in the field of oral health. This will be achieved through an ambitious systematic survey to identify various relevant resources, including clinical practice guidelines, policy statements and health technology assessments.

 The researchers will systematically search for relevant oral health documents and source organisations responsible for their development. Duplicate screening for eligibility will be conducted. 

 Calibrated data extraction at several levels (organisational, document and actionable statement level) will be carried out, and the data subject to descriptive statistical and qualitative analysis, using taxonomies described in the protocol. 

 Based on this analysis, the researchers aim to identify barriers and facilitators for the use of research evidence and decision making in these types of documents and to provide recommendations for production of future oral health guidelines.

 The researchers could consider also including the ECRI Guidelines Trust (https://guidelines.ecri.org/) as a guideline repository in their search. Keeping healthcare guidelines up-to-date is increasingly important and challenging as more guidelines become available and resources to maintain them are stretched. It would be particularly useful if the survey could comment on methodologies identified for reviewing and updating actionable statements.

 I believe this to be a novel and very valuable area of research. This proposed work is very clearly described. It has the potential to serve a crucial role in advancing understanding about the development evidence-informed oral health guidance globally and could contribute towards coordinating and standardising processes to provide more consistently developed resources.

Is the study design appropriate for the research question?

Yes

Is the rationale for, and objectives of, the study clearly described?

Yes

Are sufficient details of the methods provided to allow replication by others?

Yes

Are the datasets clearly presented in a useable and accessible format?

Yes

Reviewer Expertise:

Evidence-based healthcare.  Implementation science.

I confirm that I have read this submission and believe that I have an appropriate level of expertise to confirm that it is of an acceptable scientific standard.

# Reviewer report: Reviewer response for version 1

**DOI:** 10.5256/f1000research.154867.r216659

**Published:** 2023-10-31

**Authors:** Carolina Castro Martins-Pfeifer

**Affiliations:** 1 School of Dentistry, Federal University of Minas Gerais, Belo Horizonte, Brazil

I congratulate the authors on this systematic survey. The aim is to identify organizations developing guidelines in oral health globally, and describe the methods used to formulate the statements. The development of clinical practice guidelines may be common in medicine. However, related to oral health, some continents may be stronger in producing clinical practice guidelines than others.

 The protocol will systematically search electronic databases for organizations, guidelines and policy documents. A manual search will also be included. Paired independent reviewers will screen documents and extract data. Data will be extracted at the organizational level (such as Ministries of Health, for example) and for guideline or policy document level. The authors plan a descriptive analysis. Even if it is not the study's main purpose, I suggest the authors consider testing differences between the variables collected stratified per continent.

 In the discussion, the research plan will help to identify barriers and facilitators for using research evidence and evidence to decision frameworks in these documents. Moreover, the research could help connect a collaborative network for evidence-informed guidelines and policy documents worldwide.

 The research subject is original and important to oral health, especially due to the need to increase the high quality guidelines in all the continents. The protocol is well written. The tables showing the variables to be extracted are very helpful.

Is the study design appropriate for the research question?

Yes

Is the rationale for, and objectives of, the study clearly described?

Yes

Are sufficient details of the methods provided to allow replication by others?

Yes

Are the datasets clearly presented in a useable and accessible format?

Yes

Reviewer Expertise:

evidence-based practice and pediatric dentistry.

I confirm that I have read this submission and believe that I have an appropriate level of expertise to confirm that it is of an acceptable scientific standard.
